# Hepatitis B Surface Antigen Suppresses the Activation of Nuclear Factor Kappa B Pathway *via* Interaction With the TAK1-TAB2 Complex

**DOI:** 10.3389/fimmu.2021.618196

**Published:** 2021-02-25

**Authors:** Feiyan Deng, Gang Xu, Zhikui Cheng, Yu Huang, Caijiao Ma, Chuanjin Luo, Chen Yu, Jun Wang, Xiupeng Xu, Shi Liu, Ying Zhu

**Affiliations:** ^1^ State Key Laboratory of Virology, Modern Virology Research Center, College of Life Sciences, Wuhan University, Wuhan, China; ^2^ Department of Pathology, Tongji Hospital, Tongji Medical College, Huazhong University of Science and Technology, Wuhan, China; ^3^ Department of Clinical Laboratory, Huangshi Central Hospital, Affiliated Hospital of Hubei Polytechnic, Huangshi, China

**Keywords:** immune escape, hepatitis B surface antigen, TAK1-TAB2, phosphorylation, polyubiquitination, autophagic degradation

## Abstract

Chronic hepatitis B is a major health problem worldwide, with more than 250 million chronic carriers. Hepatitis B virus interferes with the host innate immune system so as to evade elimination *via* almost all of its constituent proteins; nevertheless, the function of HBsAg with respect to immune escape remains unclear. This study aimed to determine the role HBsAg plays in assisting HBV to escape from immune responses. We found that HBsAg suppressed the activation of the nuclear factor kappa B (NF-кB) pathway, leading to downregulation of innate immune responses. HBsAg interacted with TAK1 and TAB2 specifically, inhibiting the phosphorylation and polyubiquitination of TAK1 and the K63-linked polyubiquitination of TAB2. Autophagy is a major catabolic process participating in many cellular processes, including the life cycle of HBV. We found that HBsAg promoted the autophagic degradation of TAK1 and TAB2 *via* the formation of complexes with TAK1 and TAB2, resulting in suppression of the NF-κB pathway. The expression of TAK1, TAB2, and the translocation of NF-κB inversely correlated with HBsAg levels in clinical liver tissues. Taken together, our findings suggest a novel mechanism by which HBsAg interacts with TAK1-TAB2 complex and suppresses the activation of NF-κB signaling pathway *via* reduction of the post-translational modifications and autophagic degradation.

## Introduction

Viral hepatitis is a major public health threat, causing approximately 1 million deaths per year. Complications of chronic hepatitis led to 96% of those deaths, while 66% of chronic hepatitis cases were caused by HBV (1), despite the fact that a vaccine for HBV has been available for almost 30 years. Chronic hepatitis B virus (HBV) is carried by more than 250 million individuals, in whom it causes liver diseases ranging from asymptomatic carriage, fulminant hepatitis, chronic hepatitis, and cirrhosis to hepatocellular carcinoma (2).

HBV is a hepatotropic, non-cytopathic partially double-stranded DNA virus (3). After its entry into host cells *via* sodium taurocholate cotransporting polypeptide receptor (4), the uncoated viral genome translocated to nucleus and converted to covalently closed circular DNA, which is the transcription template for all viral RNAs (5). In the cytoplasm, viral mRNAs are translated into following viral proteins: small/middle/large surface protein (HBsAg), precore (HBeAg)/core protein (HBcAg), polymerase proteins, and the non-structural HBV X protein (HBxAg) (6).

The innate immune system serves as the first line of the host defense against virus infection, and the NF-κB signaling pathway plays an important role in it. Virus particles are recognized by pattern-recognition receptors (7–9). The canonical NF-κB signaling pathway is triggered by signals from those receptors, which active the kinase TGFβ-activated kinase 1(TAK1) (10). Activated TAK1 then phosphorylates the I kappa B kinase (IKK) complex (composed of IKKα, IKKβ, and NEMO) and IkBа, leading to the translocation of p50/p65 and the production of IFN and inflammatory cytokines (11–13).The TAK1-TAB complex has a pivotal role in the innate immune signaling pathways stimulated by virus. The TAK1 protein kinase complex is composed of its binding partners, TAB1, TAB2, and TAB3. TAB1 is constitutively bound to TAK1, While TAB2 or TAB3 are recruited after stimulation. TAB2 or TAB3 binds to K63-linked polyubiquitin chains on TRAF6 or RIP1 because of the C-terminal NZF ubiquitin-binding domain, promoting TAK1 activation (14, 15).

HBV behaves like a “stealth” virus that establishes persistent infection in hepatocytes using multiple evasion strategies to suppress host innate and adaptive immune systems (16–18). Almost all HBV proteins interfere with intracellular signal transduction pathways, such as IFN associated signaling pathways and inflammation-related pathways (19–32). HBsAg is widely known as a structure protein which is the main component of both HBV virions and subviral partials. Many studies have also reported that HBsAg works as an immune inhibitor to help HBV surviving from the snipe of host immune systems. HBsAg promotes GP73 production, which facilitates HBV replication by repressing the NF-κB signaling pathway (27). HBsAg also blocks the TLR9-interferon regulatory factor 7-IFNα signaling pathway by upregulating the expression of suppressor of cytokine signaling (28). HBsAg may directly contribute to the dysfunction of myeloid dendritic cells in patients with chronic HBV, which could be considered as a potent mechanism by which HBV escapes from immune systems (33). However, the precise molecular mechanisms by which HBV escapes from innate immune with the help of HBsAg remain unclear.

Autophagy is a major catabolic process that degrades and recycles damaged organelles and long-lived cytoplasmic macromolecules, involving in cellular processes such as maintenance of cellular homeostasis, suppression of tumor development and influence on pathogen replication (34–36). HBV infection induces autophagy mainly through the HBx protein or an HBsAg-dependent mechanism (37–39). The autophagy process was bound up with the life cycle of HBV (40). Early autophagy is required for HBV replication and envelopment, while late autophagy is associated with the degradation of cargoes, including HBV virions and proteins (such as HBsAg) (38, 41–43). We expect to know whether or not autophagy works in the process of HBsAg associated HBV immune escapes.

In the present study, we demonstrated that HBsAg specifically bound to TAK1 and TAB2, and then suppressed the polyubiquitination of TAK1-TAB2 complex and blocked TAK1 phosphorylation, leading to the inhibition the NF-κB signaling pathway. HBsAg interfered the molecular interaction between TAK1 and TAB2 by promoting autophagic degradation of TAK1 and TAB2, further inhibiting the activation of NF-κB signaling pathways.

## Materials and Methods

### Ethics Statement

Serum from 34 HBV-infected patients were collected at HuangShi Central Hospital (Hubei, China). Ten HBV-infected patients and ten healthy control subjects were recruited from Tongji Hospital (Wuhan, China). All samples were collected between 2014 and 2019. This study was approved by the institutional review board of Wuhan University, and was conducted in accordance with the principles of the Declaration of Helsinki. We acquired written informed consent from volunteers. Detailed information is described in **Supplementary Table I**.

### Cell Culture, Transfection, and Infection

Human embryonic kidney (HEK293T) cells and the human hepatoma cell line (Huh7, HepG2, HepG2.215, HepG2-hNTCP, and HepAD38 cells, purchased from the China Center Type Culture Collection) were cultured in DMEM (Gibco, Thermo Fisher Scientific, Waltham, MA, USA). Human THP-1 monocytes were cultured in RPMI 1640 medium. All media were supplemented with 10% heat-inactivated fetal bovine serum, 100 U/ml penicillin, and 100 mg/ml streptomycin in a humidified incubator with 5% CO2 maintained at 37°C. HepAD38 cells were supplemented with 10% heat-inactivated fetal bovine serum (Gibco, US), 100 U/ml penicillin, 100 mg/ml streptomycin, and 1 μg/ml adriamycin (Dox) in a humidified incubator with 5% CO_2_ maintained at 37°C.

HEK293T cells were transfected with polyethylenimine according to the manufacturers’ instructions. Huh7 and HepG2 cells were transfected with Lipofectamine 2000 (Invitrogen, US). Human THP-1 monocytes were transfected with electroporation method as described previously (44). Vesicular stomatitis virus (VSV, Indiana serotype) was provided by the China Center for Type Culture Collection. Cells were incubated with DMEM or RPMI 1640 containing SeV/VSV (MOI = 1) or equivalent PBS for indicated intervals.

### Plasmids, Antibodies, and Reagents

3×HA-TAB1, 3×HA-TAK1, 3×HA-RIG-I, and 3×HA-MyD88 were subcloned directly into PKH3-3×HA mammalian expression vectors. DsRed-TAK1 was subcloned directly into pDsRed-Monomer-N1 vector. GFP-LC3B was a gift from Professor Mingzhou Chen of Wuhan University, China. 3×Flag-HBsAg (3×Flag-HBs, genome D, Serotype ayw, expressing the full length HBsAg, large HBsAg), 3×Flag-SHBsAg, 3×Flag-MHBsAg, pBlue-HBV 1.3, Flag-TAB1, 3×HA-TAB2, 3×HA-TAB3, and ISRE/IFNβ/IFNλ1/NF-κB/IL16 luciferase reporter plasmids were previously cloned by our laboratory. Myc-Ub/K48/K63/K48R/K63R and Myc-TAK1 were cloned into pcDNA3.1 Myc-His (–) vector.

Antibodies against Flag (M185-3L), HA (M180-3), and Myc (M192-3) were purchased from Medical & Biological Laboratories. Antibodies against TAK1 (12330-2-AP), TAB2 (14410-1-AP), NF-κB p65 (10745-1-AP), NF-κB p50 (14220-1-AP), lamin A/C (10298-1-AP), β-actin (60008-1-Ig), GAPDH (60004-1-Ig), ubiquitin (10201-2-AP) were purchased from ProteinTech Group. Antibodies against p-TAK1 (4508S), p-IκBα (2859S), IκBα (4812S) were purchased from Cell Signaling Technology. Antibodies against LC3B (A19665) were purchased from Abclonal. Antibodies against HBsAg (ab9136) were purchased from Abcam. LC3B Mouse Monoclonal Antibody (9H5) and Dylight 649 goat anti-rabbit IgG (A23620-1) were purchased from Abbkine. Cy3 goat anti -mouse IgG (H+L) (E031610-01) and FITC goat anti-rabbit IgG (H+L) (E031220-01) were purchased from EarthOx. Fluorescein (FITC)-AffiniPure goat anti-horse IgG (H+L) (108–095–003) was purchased from Jackson ImmunoResearch Laboratories. MG132 and chloroquine (CQ) were purchased from TopScience.

### Harvest of HBV Inoculums and Infection of HepG2-hNTCP Cells

HepAD38 cells were cultured in DMEM/F-12 medium (Gibco, Thermo Fisher Scientific, Waltham, MA, USA) for 3 days, supplemented with 10% heat-inactivated fetal bovine serum (Gibco, US), 100 U/ml penicillin, and 100 mg/ml streptomycin in a humidified incubator with 5% CO_2_ maintained at 37°C. The supernatants of HepAD38 cells were concentrated 100-fold by ultracentrifugation as HBV inoculums. HBV stock titer (multiplicity of genome equivalents) was measured by using qPCR.

HepG2-hNTCP cells were cultured in primary hepatocytes maintenance medium (PMM) for 6 h and then inoculated with 1,000 multiplicity of genome equivalents of HBV in PMM with 4% PEG 8000 at 37°C for approximately 16 h. After the virus-containing medium were removed, cells were washed with PBS three times to remove residual viral particles and cultured in fresh PMM medium. The medium was refreshed every other day. Cells were harvested for research on 7 days after infection.

### ELISA

The supernatants of HBV infected-HepG2-hNTCP cells were collected and a standard ELISA kit was used to quantify HBsAg (Shanghai KeHua Biotech, Shanghai, China).

### qRT-PCR Analysis

Total RNA was isolated using the TRIzol reagent (Invitrogen, US) and cDNA was reverse transcribed with the TRUEscript H Minus M-MuLV Reverse Transcriptase (Aidlab Biotechnologies, China). The relative abundances of indicated mRNA were normalized to GAPDH. qRT-PCR assays were performed using the Bio-Rad CFX connect system with iTaq Universal SYBR Green Supermix (BioRad, US). All primers used for qRT-PCR assays are described in **Supplementary Table II**.

### Luciferase Assays

HepG2 cells or Huh7 cells were plated in 24-well plates, and were co-transfected with indicated plasmids when cells achieved 80% confluence. pRL-TK was used as an internal control. The luciferase activity assays were measured according to the manufacturer’s instructions of dual-luciferase reporter assay (Promega, US). The data were expressed as fold-expression normalized to pRL-TK.

### MTS Assays

HepG2 cells or Huh7 cells were seeded into 96-well plates. After culturing for 24 h, cells were transfected with 3×Flag-HBsAg or empty vector plasmids and incubated for 24 h. Next, cells were serum-starved for 12 h, and then treated with SeV (MOI = 1) for 12 h. Then, cell viability was assessed using the MTS assays. We added 20 μl Cell Titer 96^®^ AQueous One Solution Cell Proliferation Assay (Promega, US) per well. After incubating for 3 h, absorbance was measured by a microplate reader (BioTek, US) at 490 nm.

### Coimmunoprecipitation and Western Blotting Analysis

Cells were lysed in 1 ml Nonidet P-40 lysis buffer containing 20 mM Tris-HCl (pH 7.4–7.5), 150 mM NaCl, 1 mM EDTA, 1% Nonidet P-40, and 1% protease inhibitor mixture. Bio-Rad Protein Assay (BioRad, US) was used to evaluate the protein concentration. For co-immunoprecipitation, cell lysates (1–5 mg) were prewashed by adding protein A/G agarose (Pierce, US) prior to incubation on 4°C for 2 h, then IgG or indicated antibodies (2 μg) were added to supernatants for 4 h at 4°C, captured by adding the protein A/G agarose (Pierce, US) prior to incubation on 4°C for 4 h, and the precipitates were washed five times with lysis buffer containing 500 mM NaCl as mentioned above. The immune complexes were separated, subjected to SDS-PAGE, transferred to nitrocellulose membranes, blocked in skim milk and probed with the indicated antibodies.

### Nuclear Extraction

Harvested cells were washed with ice-cold PBS for three times, and then resuspended with hypotonic buffer A (10 mM Tris-HCl [pH 7.4], 5 mM MgCl_2_, 10 mM NaCl, 1 mM DTT, 10% protease inhibitor mixture) for 15 min on ice before incubation in 0.5% Nonidet P-40 on ice for 1 min. Whole cell lysates were centrifuged at 13,000 *g* for 3 min at 4°C. The nuclear extracts were pellets and the supernatants were collected as cytoplasm extracts. The nuclear extracts were resuspended with hypotonic buffer A for 10 min on ice and collected by centrifugation, after which the supernatants were removed. The pellets were resuspended in buffer B (20 mM HEPES-KOH [pH 7.9], 1.5 mM MgCl_2_, 0.5 mM NaCl, 1 mM DTT, 0.2 mM EDTA, 1% Nonidet P-40, 10% protease inhibitor mixture, 1% Nonidet P-40), vortexed for 15 s, and incubated on ice for 30 min, centrifuged at 13,000 *g* for 30 min together with the cytoplasm extracts; then, the nuclear and the cytoplasm protein-containing supernatants were collected. All fractions were stored at –80C until use. The indicated proteins were examined using western blot.

### Immunofluorescence Microscopy

For confocal analysis, HepG2 cells were plated on collagen incubated in 14-mm confocal dishes, transfected with indicated plasmids and treated with indicated reagents. Cells were washed with 1× PBS three times, fixed with 4% paraformaldehyde, permeabilized with 0.3% Triton X-100, and blocked with PBS containing 3% BSA for 1 h at room temperature. Then, the cells were immunostained with the indicated primary Abs overnight at 4°C followed by incubation with the relevant dye-conjugated secondary Abs at 37°C for 1 h. The nuclei were stained with 4’,6-diamidino-2-phenylindole for 3 min at 37°C. The cells were imaged using a fluorescence microscope (Leica, Germany) with 100× objective lens. The analysis for colocalization was conducted with Image J.

Liver tissues for immunofluorescence assays were collected from patients as described above. LC3 and TAK1 proteins were examined and visualized using anti-LC3B 9H5 and anti-TAK1 (12330-2-AP), respectively.

### Immunohistochemistry

Liver tissues were collected from patients as mentioned above. The expression of HBsAg, TAK1, TAB2, p50, p65 in liver tissue was visualized using immunohistochemical staining of thin liver tissue sections using indicated antibodies. The analysis for the expression of protein was conducted with Image J.

### Statistical Analysis

All data were expressed as mean values ± standard deviation. Statistical analyses were performed using Graph Pad Prism software (GraphPad Software Inc., La Jolla, CA, USA). The two-tailed Student’s t-test was used to determine significant differences. Differences were considered statistically significant when P < 0.05.

## Results

### HBsAg Suppresses the Antiviral Innate Immune Response

**Figure 1:**
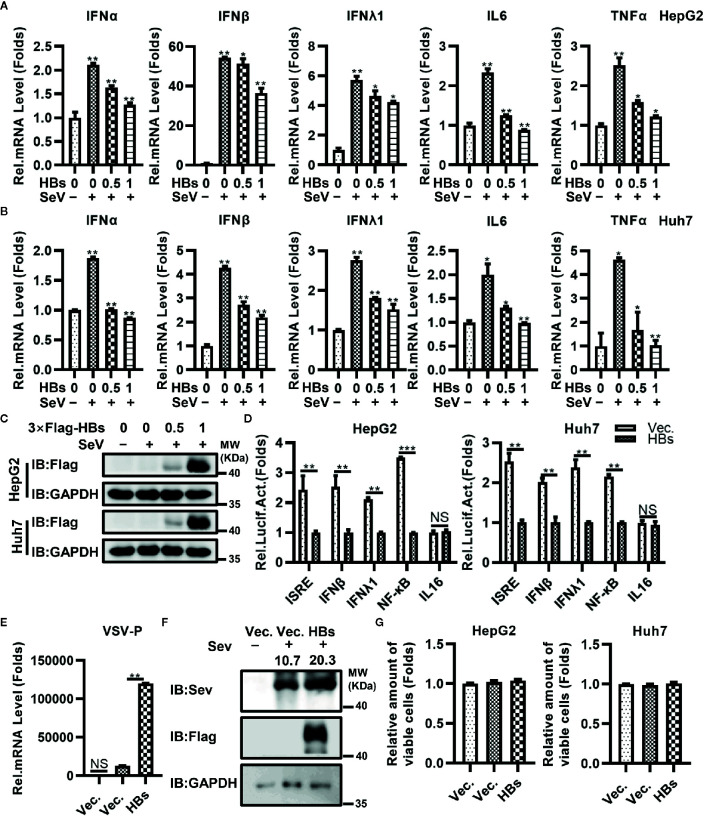
HBsAg down-regulated the antiviral innate immune response in hepatic cells. **(A)** HepG2 cells and **(B)** Huh7 cells were transfected with different doses (0, 0, 0.5, 1 μg) of HBsAg (HBs) expression plasmids (3×Flag-HBsAg), the total amount of plasmids was adjusted to 1 μg with empty vector. At 24 h post-transfection, the cells were serum starved for 12 h. Cells were mock infected or infected with SeV (MOI = 1) for 12 h, and then harvested for qRT-PCR analysis. **(C)** HepG2 or Huh7 cells were transfected with different doses (0, 0, 0.5, 1 μg) of 3×Flag-HBs expression plasmids, the total amount of plasmids was adjusted to 1 μg with empty vector. At 24 h post-transfection, the cells were serum starved for 12 h. Cells were mock infected or infected with SeV (MOI = 1) for 12 h, and then harvested for western blots. **(D)** ISRE/IFNβ/IFNλ1/NF-κB/IL16 luciferase reporter plasmids (0.3 μg) were co-transfected into HepG2 cells or Huh7 cells with HBs expression plasmids/vector plasmids (0.2 μg) and pRL-TK (0.1 μg). At 24 h post transfection, the cells were serum starved for 12 h. Next cells were infected with SeV (MOI = 1) for 12 h, and then harvested for luciferase activities determination. **(E)** HBs expression plasmids or vector plasmids (0.5 μg) were transfected into HepG2 cells. At 24 h after transfection, cells were mock infected or infected with vesicular stomatitis virus (VSV) (MOI = 1) for 12 h. The mRNA levels of VSV P protein were determined by qRT-PCR. **(F)** HBs expression plasmids or vector plasmids (1 μg) were transfected into HepG2 cells, at 24 h post-transfection, cells were mock infected or infected with SeV (MOI = 1) for 24 h, and then harvested for western blot. **(G)** HBs expression plasmids or vector plasmids (0.1 μg) were transfected into HepG2 cells and Huh7 cells. At 24 H post transfection, the cells were serum starved for 12 h. cells were infected with SeV (MOI = 1) for 12 h, and then conducted the MTT assays. All experiments were repeated three times with consistent results. The graphs show the means ± SD, n = 3 (***P < 0.01; **P < 0.01; *P < 0.05), NS., not significant.

To investigate the potential role of HBsAg in the immune escape of HBV, we constructed the 3×Flag-HBsAg (3×Flag-HBs, genome D, Serotype ayw, expressing the full length HBsAg). HBsAg (HBs) as mentioned below is large HBsAg. We measured expression of IFNα, IFNβ, IFNλ1, IL6, and TNFα at the mRNA level in both HepG2 and Huh7 cell lines transfected with increasing dose of 3×Flag-HBs using qRT-PCR testing, triggered with SeV. As shown in **Figures 1A–C**, overexpression of HBs reduced the expression of IFNα, IFNβ, IFNλ1, IL6, and TNFα in HepG2 and Huh7 cells in a dose-dependent manner. We next transfected 3×Flag-HBsAg into macrophages induced from THP-1 *via* electroporation, triggered with SeV. HBsAg reduced the expression of IFNα, IFNβ, IFNλ1, IL6, and TNFα in macrophages induced from THP-1 (**Supplementary Figure 1A**). All kinds of HBsAg decreased the virus-trigged expression of IFNβ and TNFα (**Supplementary Figure 1B**). We performed reporter assays in both HepG2 and Huh7 cell lines infected with SeV, and found that HBsAg significantly inhibited the activation of NF-κB promoters. The activation of ISRE, IFNβ and IFNλ1 promoters was also inhibited by HBsAg, while almost had no influence on the IL16 promoter (negative control) in both HepG2 and Huh7 cells (**Figure 1D**). 3×Flag-HBs or control vector were transfected into HepG2 cells, at 24 h post-transfection, cells were mock infected or infected with VSV or SeV and harvested for qRT-PCR analysis or western blot. Overexpression of HBsAg facilitated both the replication of VSV and SeV in HepG2 cells (**Figures 1E, F**). 3×Flag-HBs or control vector were transfected into HepG2 cells and Huh7 cells for 24 h. Cells were harvested to conduct the MTT assays after infected with SeV for 12 h. HBsAg showed no cytotoxicity in either HepG2 or Huh7 cells (**Figure 1G**). Taken together, these data suggest that HBsAg inhibits the antiviral innate immune response in host cells.

### HBsAg Specifically Interacts With the TAK1-TAB2 Complex

**Figure 2:**
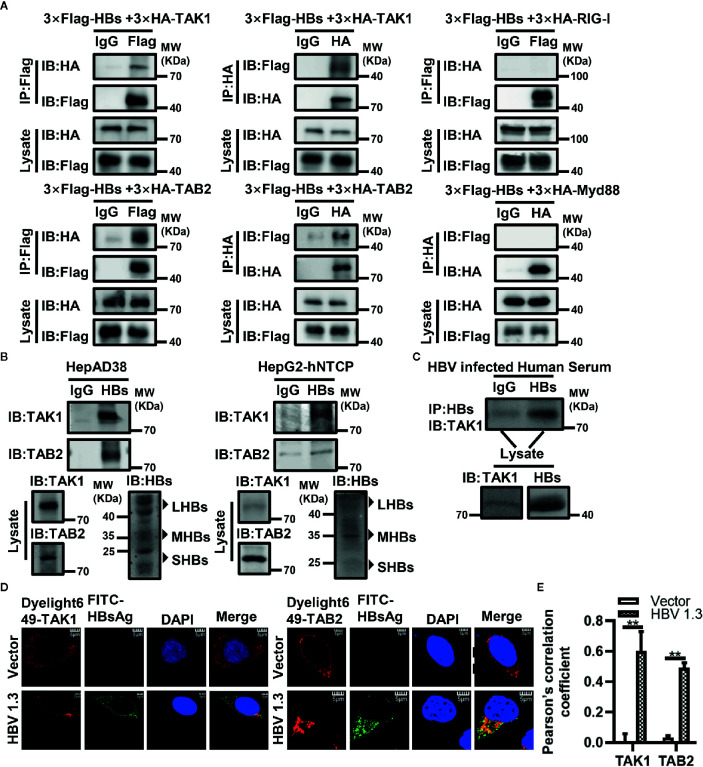
HBsAg interacts with TAK1 and TAB2 specifically *in vitro* and *in vivo*. **(A)** 3×Flag-HBs or vector plasmids were co-transfected into human embryonic kidney (HEK293T) cells with 3×HA-TAK1/TAB2/RIG-I/Myd88, at 48 h post transfection, cells were harvested for Co-IP assays. **(B)** HepAD38 cells were seeded in 10-cm wells, and then harvested for Co-IP assays at 48 h later. HepG2-hNTCP were seeded in 10-cm wells with primary hepatocytes maintenance medium (PMM) for 6 h, and then infected with 1000 multiplicity of genome equivalents of HBV in PMM with 4% PEG 8000 at 37°C for approximately 16 h. After the virus-containing medium were removed, cells were washed with PBS three times to remove residual viral particles and cultured in fresh PMM medium. The medium was refreshed every other day. Cells were harvested for Co-IP assays on 7 days after infection. We divided the cell lysate of HepAD38 cells or HBV infected HepG2-hNTCP cells into three. One of which was immunoprecipitated with IgG, one was with anti-HBsAg antibody, and the other was prepared as lysate for western blot. Proteins were immunoblotted with indicated antibody. **(C)** The serum of HBV infected patients was diluted 20 times for Co-IP assays. We divided the diluted serum into three aliquots. One of which was immunoprecipitated using IgG, one was incubated with anti-HBsAg antibody, and the other was used for western blot. Proteins were immunoblotted with indicated antibodies. We found that HBsAg interacted specifically with TAK1 *in vivo*. **(D)** HepG2 cells were plated on collagen incubated in 14-mm confocal dishes, and then HBV1.3 expression plasmids or control vector were transfected into HepG2 cells for 24 h to conduct confocal microscopy assays. HBsAg were stained with fluorescein (FITC)-AffiniPure goat anti-horse IgG (H+L). TAK1 and TAB2 were stained with Dylight 649 goat anti-rabbit IgG. The nuclei were stained with 4’,6-diamidino-2-phenylindole (DAPI). The cells were imaged using a fluorescence microscope (Leica, Germany) with 100× objective lens. Scale bar = 5 μm. **(E)** We analyze the level of colocalization between HBsAg with TAK1/TAB2 by using Image J and GrapPad Prism 8. The graphs show the means ± SD, n = 3 (**P < 0.01).

To determine which protein in the antiviral innate immune pathway is the target of HBsAg, we conducted Co-IP assays. Indicated plasmids were co-transfected into HEK293T cells for 48 h, and then harvested for immunoprecipitation and immunoblot with indicated antibodies. We found that HBsAg interacted specifically with TAK1 and TAB2 in HEK293T cells, but not with RIG-I and Myd88 (**Figure 2A**). Interestingly, HBsAg did not interact with TAB1 or TAB3 (**Supplementary Figure 2A**), which also belong to TAK1-TAB complex. Small HBsAg and middle HBsAg interacted with TAK1 *in vitro* (**Supplementary Figure 2B**). We harvested HepAD38 cells and HBV infected HepG2-hNTCP cells for Co-IP assays. We found endogenous interactions of HBsAg with TAK1 and TAB2 in HepAD38 cells and HBV infected HepG2-hNTCP cells (**Figure 2B**). We diluted the serum of HBV-infected patients 20 times for Co-IP assays, immunoprecipitated with anti-IgG or anti-HBsAg and immunoblotted with indicated antibodies. In the serum of HBV-infected patients, we found that TAK1 interacted with HBsAg specifically (**Figure 2C**). HBV 1.3 expression plasmids or empty vector were transfected into HepG2 cells for 24 h to conduct confocal microscopy (**Figure 2D**). We assessed the level of colocalization between HBsAg with TAK1/TAB2 by Pearson’s correlation coefficient calculated *via* Image J (**Figure 2E**). Confocal imaging analysis cells revealed that HBsAg colocalized with both TAK1 and TAB2 *in vitro*. These data suggest that HBsAg interacts with TAK1-TAB2 complex specifically *in vitro* and *in vivo*.

### HBsAg Suppresses the Polyubiquitination of the TAK1-TAB2 Complex

**Figure 3:**
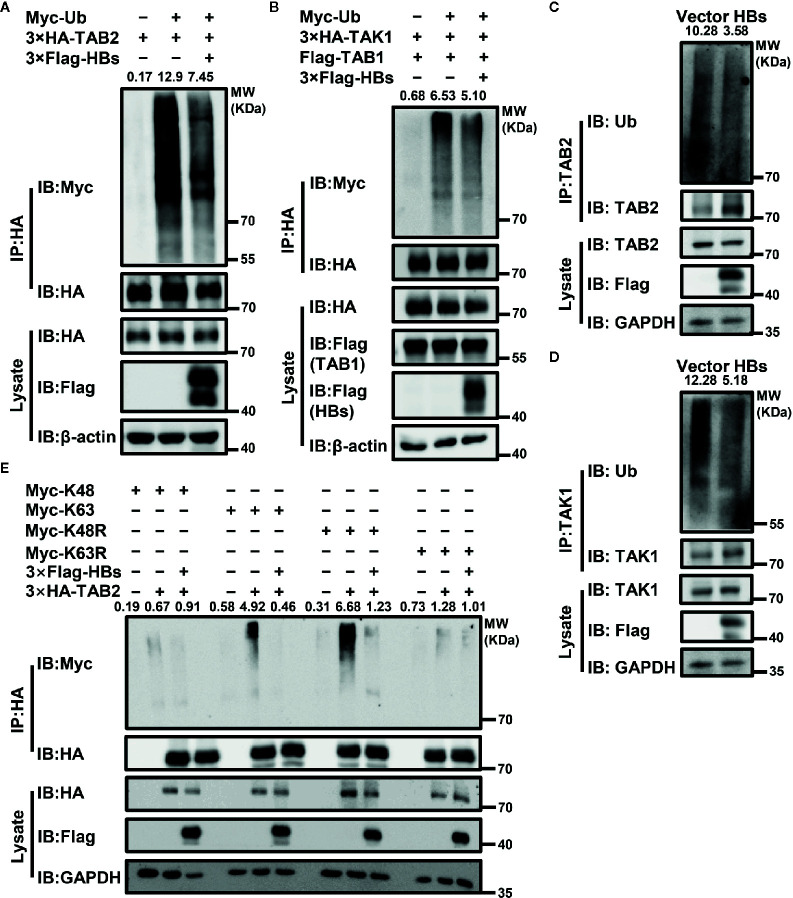
HBsAg suppresses the polyubiquitination of TAK1-TAB2 complex. **(A)** 3×Flag-HBs (HBsAg) or vector plasmids were co-transfected into Huh7 cells with 3×HA-TAB2 and Myc-Ub for 36 h, and then cells were stimulated with virus for 12 h. The cell lysate was immunoprecipitated and immunoblotted using indicated antibody, and then analyzed the ubiquitination abundance of corresponding protein. **(B)** 3×HA-TAK1, Flag-TAB1, Myc-Ub were co-transfected into Huh7 cells with 3×Flag-HBs or vector plasmids for 36 h. Cells were then treated with virus for 12 h, followed by ubiquitination assays. **(C, D)** The levels of endogenous TAB2 and TAK1 ubiquitination in HBsAg-overexpressing Huh7 Cells infected with virus. HBs expression plasmids (3×Flag-HBs) or vector plasmids were transfected into Huh7 cells for 36 h, and then cells were stimulated with virus for 12 h, followed by ubiquitination assays. The same samples were used in **(C, D)**. **(E)** 3×HA-TAB2, Myc-K48/K63/K48R/K63R plasmids were co-transfected with 3×Flag-HBs or control vector into Huh7 cells for 36 h as indicated, and then cells were stimulated with virus for 12 h, followed by ubiquitination assays. We use the Image J for the abundance analyses.

Ubiquitination is essential to the activation of the TAK1-TAB2 complex. Therefore, we next determined whether the expression of HBsAg affected the ubiquitination of the TAK1-TAB2 complex. Indicated plasmids were co-transfected with Myc-Ub into Huh7 cells. Cells were stimulated with virus, and then harvested for Co-IP assays with indicated antibodies to analyze the ubiquitination abundance of corresponding protein. As shown in **Figures 3A, B**, co-expression of HBsAg reduced the ubiquitination of TAB2 and TAK1 in the presence of exogenous ubiquitin. We next tested the level of endogenous TAB2 and TAK1 ubiquitination in HBsAg-overexpressing Huh7 cells infected with virus. The polyubiquitination of TAB2 (**Figure 3C**) and TAK1 (**Figure 3D**) decreased in the presence of HBsAg in virus infected Huh7 cells. The same samples were used in **Figures 3C**, **D**. To determine what type of ubiquitination modification in TAB2 was blocked by HBsAg, we then co-transfected Myc-K48/K63/K48R/K63R, 3×HA-TAB2, 3×Flag-HBs or empty vector as indicated in **Figure 3E**. Here, K48R, or K63R means that only Lysine48 or Lysine63 residue was mutated to arginine, K63 or K48 means only Lysine63 or Lysine48 residue remains unchanged while the other lysines were all mutated to arginines. The results showed that HBsAg weakened the K63 and K48R-linked polyubiquitination, while the K48 and K63R remained unaltered. It indicated that HBsAg restrained K63-linked, but not K48-linked polyubiquitination of TAB2. When the K48 residue of the wild-type ubiquitin chain was mutated to R, with K63 still present, we found that HBsAg could still downregulate the K48R-mediated ubiquitination of TAB2, which further proves that HBsAg mainly regulates the ubiquitination of TAB2 at K63 site. These results suggest that HBsAg down-regulates the polyubiquitination of the TAK1-TAB2 complex, especially the K63-linked polyubiquitination of TAB2.

### HBsAg Inhibits the Activation of NF-κB Signaling Pathway by Blocking TAK1 Phosphorylation

**Figure 4:**
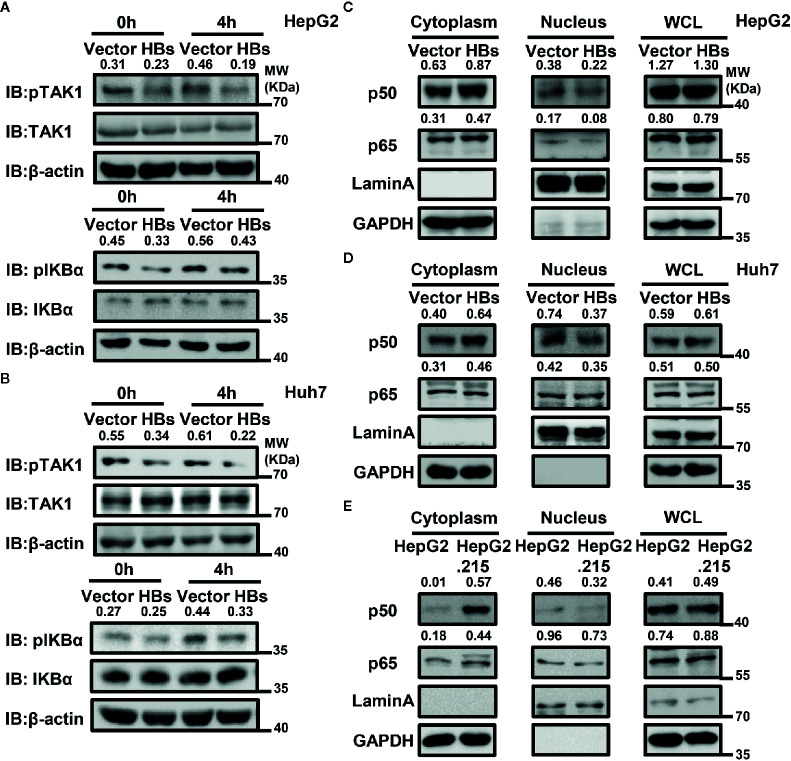
HBsAg inhibits virus-triggered nuclear factor kappa B (NF-kB) activation. **(A, B)** HBsAg suppresses the phosphorylation of TAK1 and IKBα in HepG2 and Huh7 cells. HepG2 **(A)** or Huh7 **(B)** cells were transfected with vector plasmid or the HBs (3×Flag-HBsAg) expression plasmid for 24 h, then infected with SeV (MOI = 1) for indicated times, then harvested and immunoblotted with the indicated antibodies. **(C, D)** HBsAg blocked NF-κB subunits translocation from cytoplasm into the nucleus in HepG2 and Huh7 cells. HepG2 **(C)** or Huh7 **(D)** cells were transfected with vector plasmid or the HBs (3×Flag-HBsAg) expression plasmid for 24 h; HepG2 and HepG2.215 **(E)** were plated on collagen-coated six-well plate for 24 h. Then, cells were infected with SeV (MO I= 1) for 12 h. The whole cell lysates, the cytosolic and nuclear extracts were prepared as indicated in materials and methods, followed by western blot with indicated antibodies. GAPDH and LaminA were used as cytosolic and nuclear markers respectively. We use the Image J for the protein abundance analyses. All experiments were repeated three times with consistent results, and we showed the most representative results.

In addition to ubiquitination, phosphorylation is also of great importance in the activation of the NF-κB signaling pathway. We next examined the effect of HBsAg on SeV-triggered TAK1 phosphorylation and subsequent NF-κB translocation and activation. An overexpression system was used in HepG2 and Huh7 cells, and cells were harvested for western blot with indicated antibodies at indicated time after SeV infection. As shown in the **Figure 4A, B**, the phosphorylation of TAK1 and IKBα was blocked in the presence of HBsAg in HepG2 and Huh7 cells. The phosphorylation of TAK1 and IKBα is the key for NF-κB translocation. We then performed a nuclear extraction experiment in HepG2 and Huh7 cells to determine whether HBsAg affected NF-κB translocation. HBsAg expression noticeably reduced virus-triggered translocation of NF-κB subunits p50 and p65 from the cytosol to the nucleus in HepG2 and Huh7 cells, whereas the total expression of proteins was not affected (**Figure 4C, D**). We harvested both HepG2 and HepG2.215 cells to conduct a nuclear extraction experiment for comparing the translocation of p50 and p65. The translocation of p50 and p65 was also inhibited in HepG2.215 cells compared with in HepG2 cells (**Figure 4E**), which implied that NF-κB signaling pathway was suppressed in the presence of HBV. These findings suggest that HBsAg blocks the translocation of p50 and p65 from the cytosol to the nucleus by suppressing the phosphorylation of TAK1 and IKBα.

### HBsAg Interferes With the Interaction Between TAK1 and TAB2 Through Autophagic Degradation

**Figure 5:**
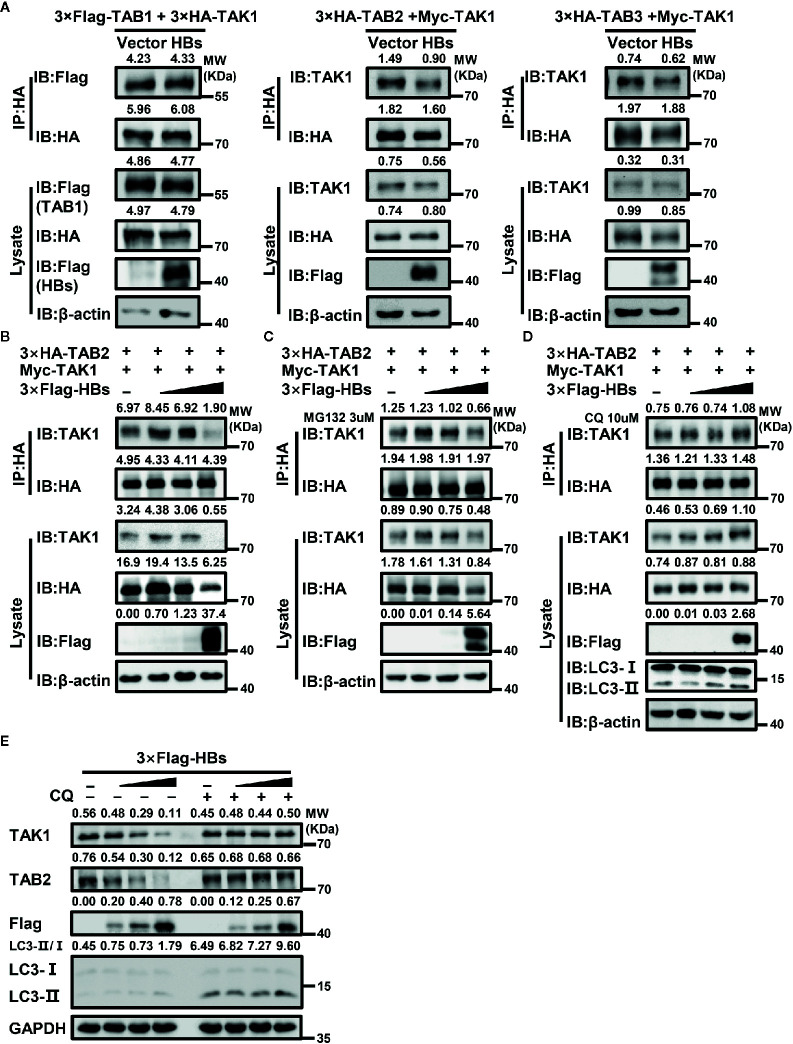
HBsAg suppresses TAK1-TAB2 interaction through autophagic degradation. **(A)** HBs expression plasmids (3×Flag-HBsAg) or vector plasmids were co-transfected into human embryonic kidney (HEK293T) cells with indicated plasmids for 48 h, and then cells were harvested for Co-IP and western blot assays using indicated antibodies. **(B, C, D)** CQ, but not MG132, reversed the suppression effect of HBsAg on the interaction between TAK1 and TAB2. HEK293T cells were co-transfected with 3×HA-TAB2 (3 μg), Myc-TAK1 (3 μg) and increasing amounts (0, 0.12, 0.6, 3 μg) of 3×Flag-HBs (HBsAg), the total amount of plasmids was adjusted to 10 μg with empty vector. At 24 h post co-transfection, cells were serum starved for 12 h and treated without **(B)** or with MG132 (3 μM) **(C)**/CQ (10 μM) **(D)** for 24 h, then harvested for Co-IP and western blot assays with indicated antibodies. **(E)** HBsAg facilitated the degradation of TAK1 and TAB2 in a dose-dependent manner in HepG2 cells, and the effect disappeared with the treatment of CQ. Increasing amount of 3×Flag-HBs (0, 0.35, 1, 3 μg) were transfected into HepG2 cells for 24 h. The total amount of plasmids was adjusted to 3 μg. Next cells were treated with or without CQ (10 μM) for 24 h, and then harvested for Co-IP and western blot assays.

HBsAg interacts with both TAK1 and TAB2. The combination of TAK1-TAB2 plays a crucial part in the activation of the NF-κB signaling pathway. We determined whether HBsAg may interfere with the formation of the TAK1-TAB complex. At 48 h after co-transfection of indicated plasmids into HEK293T cells, we conducted a Co-IP assay. HBsAg interfered with the interaction of TAK1 and TAB2, but not TAB1 and TAB3 (**Figure 5A**). Next, HEK293T cells were co-transfected with 3×HA-TAB2, Myc-TAK1 and increasing dose of 3×Flag-HBs, the total amount of plasmids was adjusted to 10 μg with empty vector. At 48 h post-transfection, cells were harvested for Co-IP and western blot assays with indicated antibodies. We found that HBsAg disrupted the TAK1-TAB2 interactions in a dose-dependent manner (**Figure 5B**).

Interestingly, with the increase of HBsAg’s expression in the whole cell lysate, protein levels of TAK1 and TAB2 decreased (**Figure 5B**). However, mRNA levels of TAK1 and TAB2 made no difference with or without the presence of HBsAg (**Supplementary Figure 1C**). Autophagy and proteasome degradation are the two major reasons for intercellular protein degradation (45). Next, we repeated the competitive Co-IP experiments (shown in **Figure 5B**) with the treatment of MG132 (a proteasome inhibitor) or chloroquine (CQ, an autophagy inhibitor). We found that CQ (**Figure 5D**), but not MG132 (**Figure 5C**), inhibited the degradation of TAK1 and TAB2 caused by the expression of HBsAg, and the interference of HBsAg on TAK1-TAB2 interaction disappeared. We next determined whether protein levels of endogenous TAK1 and TAB2 in HepG2 cells were affected by the expression of HBsAg. We transfected increasing dose of 3×Flag-HBs into HepG2 cells for 24 h, and then cells were treated with or without CQ for 24 h. Next, we collected cells for western blot analysis. As shown in **Figure 5E**, HBsAg facilitated the degradation of endogenous TAK1 and TAB2 in a dose-dependent manner in cells without the treatment of CQ, whereas increasing HBsAg levels made no difference in the expression of TAK1 and TAB2 in cells with the treatment of CQ. The value of LC3II/I increased with HBsAg in a dose-dependent manner in HepG2 cells with or without the treatment of CQ (**Figure 5E**), and HBsAg promoted the puncta formation of LC3B (**Supplementary Figure 3**), which indicated that HBsAg induced autophagy. These results suggest that HBsAg would promote the autophagic degradation of TAK1 and TAB2.

**Figure 6:**
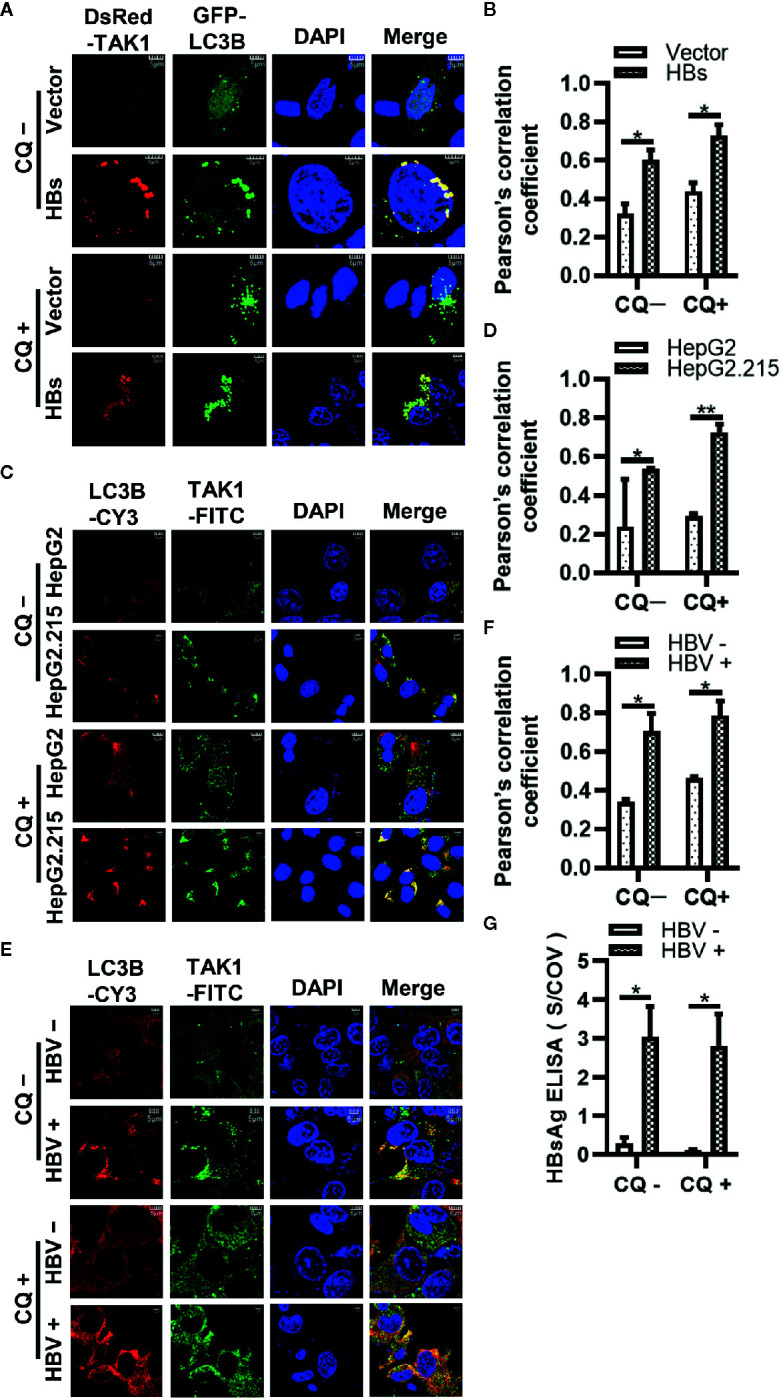
The colocalization of TAK1 and LC3B in the presence of HBsAg is increased upon CQ treatment. **(A)** DsRed-TAK1, GFP-LC3B were co-transfected into HepG2 cells with HBs expression plasmids (3×Flag-HBsAg) or empty vector for 24 h; **(C)** HepG2 and HepG2.215 cells were plated on collagen incubated in 14-mm confocal dishes for 24 h; **(E)** HepG2-hNTCP cells were cultured in collagen-coated 14-mm confocal dishes with primary hepatocytes maintenance medium (PMM) for 6 h, infected with HBV for 4 days; Then treated with or without CQ (10 μM) for 24 h. LC3B were stained Cy3 goat anti-mouse IgG (H+L) and TAK1 were stained with FITC goat anti-rabbit IgG (H+L). The nuclei were stained with DAPI before observation using confocal microscopy. Scale bar = 5 μm. **(B, D, F)** We assessed the level of colocalization by Pearson’s correlation coefficient through using Image J. **(G)** The supernatant of HepG2-hNTCP on 5 days after HBV infection were collected for HBsAg ELISA analysis. The graphs show the means ± SD, n = 3 (**P < 0.01; *P < 0.05).

To strengthen the evidence that HBsAg affects the autophagic degradation, HepG2 cells were co-transfected GFP-LC3B, DsRed-TAK1, and Vector plasmid or 3×Flag-HBs for 24 h, followed by the treatment of 10 μM CQ for 24 h. Next, the colocalization of TAK1 and LC3B was further analyzed using confocal microscopy and Image J. TAK1 and LC3B colocalized in the presence of HBsAg, which was increased upon CQ treatment in HepG2 cells (**Figures 6A, B**). Furthermore, we conducted the Immunofluorescence staining of TAK1 and LC3B in both HepG2 and HepG2.215 cells with or without treatment of CQ, the results also showed that the colocalization of TAK1 and LC3B was significantly increased in HepG2.215 cells, which was more obvious in HepG2.215 cells with the treatment of CQ (**Figures 6C, D**). Similar results also appeared in HBV positively-infected HepG2-hNTCP cells, compared to HBV negatively infected HepG2-hNTCP cells (**Figures 6E, F**). The supernatants of HepG2-hNTCP cells were collected for HBsAg ELISA analysis to determine whether or not HBV infected HepG2-hNTCP cells successfully (**Figure 6G**). These findings suggest that HBsAg promotes the degradation of TAK1 with the help of autophagic degradation, which might lead to the reduction of the interaction between TAK1 and TAB2.

### Down-Regulated Expression of TAK1 and TAB2 and Activation of NF-κB in Liver Biopsy Tumor Tissues From HBV-Infected Patients

**Figure 7:**
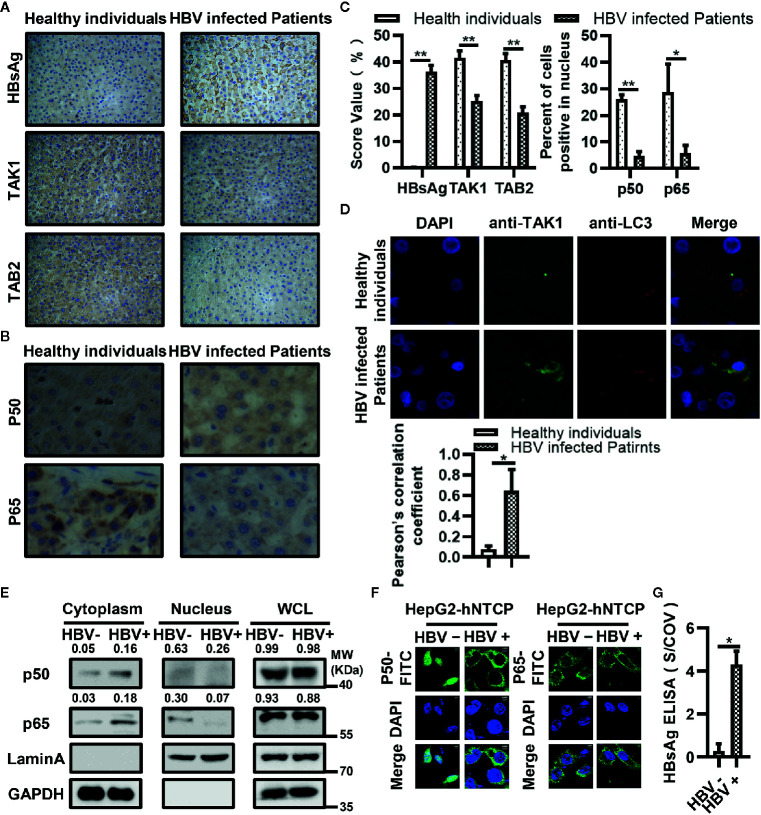
Down-regulated expression of TAK1 and TAB2 and activation of nuclear factor kappa B (NF-κB) in bioptic liver tissues from hepatitis B virus (HBV)-infected patients or HBV infected HepG2-hNTCP cells. **(A)** Immunohistochemical staining of HBsAg, TAK1, and TAB2 in bioptic liver tissues from the healthy individuals and HBV-infected patients. **(B)** Immunohistochemical staining of NF-κB subunits (p50, p65) in bioptic liver tissues from the healthy individuals and HBV-infected patients. The magnification times are 400. **(C)** The abundance of HBsAg/TAK1/TAB2 was assessed by score values after analyzed using Image J. We used the percent of cells p50/p65 located in nucleus to total cell amounts to show the p50/p65 nucleus translocation level. **(D)** Immunofluorescence in bioptic liver tissues was performed to determine the colocalization of TAK1 and LC3. **(E)** HepG2-hNTCP cells were cultured in collagen-coated six-well plates for 6 h, inoculated with 1,000 multiplicity of genome equivalents of hepatitis B virus (HBV) in primary hepatocytes maintenance medium (PMM) with 4% PEG 8000 at 37°C for approximately 16 h, removed the virus-containing medium, washed with PBS three times and cultured in fresh PMM medium. The medium was refreshed every other day. Cells were harvested for nuclear extraction on 7 days after infection. **(F)** HepG2-hNTCP cells were plated on collagen incubated in 14-mm confocal dishes with PMM for 6 h, then inoculated with 1,000 multiplicity of genome equivalents of HBV in PMM with 4% PEG 8000 at 37°C for approximately 16 h, removed the virus-containing medium, washed with PBS three times and cultured in fresh PMM medium. The medium was refreshed every other day. Cells were analyzed the translocation of p50 and p65 through immunofluorescent staining on 5 days after infection. P50 and p65 were stained with FITC goat anti-rabbit IgG (H+L), and the nuclei were stained with DAPI before observation using confocal microscopy. Scale bar = 5 μm. **(G)** The supernatant of HepG2-hNTCP on 5 days after HBV infection were collected for HBsAg ELISA analysis. The graphs show the means ± SD, n = 3 (**P < 0.01; *P < 0.05).

We selected 10 liver biopsy samples from both healthy individuals and HBV-infected patients. We first measured the differences in expression levels of HBsAg, TAK1 and TAB2 in each group. We conducted the semiquantitative analysis at the protein level using Image J. The results of immunohistochemical analysis showed that the protein levels of TAK1 and TAB2 in liver biopsy tissues from HBV-infected patients were lower than those in tissues from healthy individuals (**Figures 7A, C**). The translocation of the NF-κB subunits p50 and p65 from the cytosol to the nucleus indicates NF-κB signaling pathway activation; therefore, we then measured protein levels of p50 and p65 in both groups using immunohistochemistry. As shown in **Figures 7B, C**, levels of p50 and p65 internuclear from liver biopsy tissues of HBV-infected patients were also lower than those in tissues from healthy individuals, suggesting that the activation of NF-κB was suppressed in liver biopsy tissues from HBV-infected patients. We further confirmed that TAK1 was colocalized with LC3 in liver tissues from HBV-infected patients using immunofluorescence (**Figure 7D**). Moreover, we detected the translocation of p50 and p65 in HBV infected or not HepG2-hNTCP cells by nuclear extraction (**Figure 7E**) and immunofluorescence staining analysis (**Figure 7F**). Both p50 and p65 were inhibited from translocation from the cytosol to nucleus in HBV-infected HepG2-hNTCP cells. The supernatants of HBV infected or mock infected HepG2-hNTCP cells were collected for HBsAg ELISA analysis to determine whether or not HBV infected HepG2-hNTCP cells successfully (**Figure 7G**). Taken together, the data suggest that it is possible for HBsAg to promote the autophagic degradation of TAK1 and TAB2 and to inhibit the activation of the NF-κB signaling pathway in the context of HBV infection.

## Discussion

Chronic HBV infection is a global public health threat; millions of people suffer chronic infection with the virus (1). Previous studies have shown that HBV behaved like a “stealth” virus that establishes persistent infection in hepatocytes (16–18). Whether or not HBV stimulates and interferes with innate immune responses has been a matter of controversy. Previous studies showed that RIG-I senses HBV pregenomic RNA to induce interferon production (46), while HBx and PreS2 activate the NF-κB signaling pathway (47, 48). However, expression levels of many innate immune-related genes remain low (49) and NF-κB activity returns to a normal level (50), while infections continued and HBV replication increased. Several studies have suggested that HBV have developed strategies to escape from innate immune responses system (19–32). In addition to the persistence of covalently closed circular DNA, HBV DNA integrated into the host genome was another barrier to eliminating HBV (51). Wooddell et al. found that integrated HBV DNA was sufficiently intact to support translation of viral proteins, including HBsAg (52). Our previous study indicated that HBsAg interacted directly with MVP to break the interaction between MVP and MyD88, inhibiting the induction of type-I IFN (53). Fanny et al. found that chronic HBV infection suppressed intrahepatic immune responses, which were more intense at high levels of HBsAg (54). All these previous studies suggested that HBV is transiently sensed by the innate immune system but develops some strategies to escape from immune system, and HBsAg plays an important role in it. However, the specific molecular mechanisms by which this occurs remain obscure. In the present study, we demonstrated a novel mechanism by which HBsAg interacts with TAK1-TAB2 complexes and suppresses the activation of the NF-κB signaling pathway.

The NF-κB signaling pathway forms an integral part in the host defense against viral infection. In the present study, we found that activation of the NF-κB signaling pathway was significantly suppressed by the overexpression of HBsAg in a dose-dependent manner (**Figure 1**). We investigated which protein was the potential target of HBsAg, and discovered that HBsAg interacted specifically with TAK1 and TAB2 *in vitro* and *in vivo*, but not TAB1 and TAB3, using co-immunoprecipitation assays. In the HBV infected HepG2-hNTCP cells and serum of HBV-infected patients collected from Huangshi Central Hospital, we found that TAK1 interacted with HBsAg specifically, verifying the existence of the interaction between TAK1 and HBsAg in humans. Confocal microscopy assays further confirmed the possibility of colocation of HBsAg with TAK1-TAB2 complex (**Figure 2**).

The TAK1-TAB complex is the key to regulating the activation of the NF-κB signaling pathway. Deletion of TAK1 from hepatocytes resulted in spontaneous development of hepatocellular carcinoma (55, 56). Another study showed that TAK1 inhibited HBV replication at the viral transcription level (57). In our previous study, we found that HBV stimulated fibronectin promoted HBV expression and replication *via* interaction with TAK1-TAB complex and suppression of the NF-κB signaling pathway (58). K63-linked polyubiquitination of upstream proteins recruits TAB2 or TAB3, and then binds to TAK1, which allows autophosphorylation-dependent activation of TAK1 to occur. Both polyubiquitination and phosphorylation are of great importance in the activation of TAK1 (15). The kinase activity of TAK1 is required for TAK1-mediated inhibition of HBV replication and gene expression in hepatocytes (57). Therefore, we next measured the effect of HBsAg on the polyubiquitination and phosphorylation of TAK1-TAB2 complex. Both exogenous and endogenous ubiquitination of TAK1 and TAB2 were significantly reduced in the presence of HBsAg. K63, but not K48-linked polyubiquitination of TAB2 was inhibited by HBsAg (**Figure 3**). HBsAg also inhibited the ubiquitination of NEMO (**Supplementary Figure 2C**), a key protein in the IFN-induction pathway. Phosphorylation of TAK1 and IKBα was also blocked by HBsAg (**Figures 4A, B**). We further found that HBsAg significantly inhibited the SeV-triggered translocation of the NF-κB subunits p50 and p65 from the cytosol to the nucleus in HepG2 and Huh7 cells, whereas the total expression of proteins was not affected (**Figures 4C, D**). Both p50 and p65 accumulated more in cytosol in HepG2.215 cells than HepG2 cells (**Figure 4E**), which implied that NF-κB signaling pathway were indeed suppressed in HBV persistent replication cell lines. Moreover, we confirmed the inhibitory role of HBsAg in activation of NF-κB signaling pathway in clinical samples and HBV infected HepG2-hNTCP cells (**Figure 7**). Taken together, these findings suggest that HBsAg inhibits the activation of NF-κB signaling pathway through inhibition of the phosphorylation of TAK1 and the polyubiquitination of TAK1 and TAB2.

The formation of TAK1-TAB complex is required for the activation of TAK1 (17). We then tested whether the interaction between TAK1 and TAB2 was inhibited by the presence of HBsAg. We found that HBsAg weakened the interaction of TAK1 and TAB2, but not of TAB1 or TAB3, in a dose-dependent manner (**Figures 5A, B**). Interestingly, protein levels of TAK1 and TAB2 decreased with the increase of HBsAg expression in whole cell lysates (**Figure 5B**), which also occurred in clinical liver tissues (**Figures 7A, C**); nevertheless, mRNA levels of TAK1 and TAB2 did not change in the presence of HBsAg in HepG2 Cells (**Supplementary Figure 1C**). We next sought for the mechanism of TAK1 and TAB2 degradation in the presence of HBsAg.

Autophagy and the ubiquitin-proteasome system constitute the two major intercellular degradative mechanisms (45). In addition to proteasome degradation, selective autophagy may contribute to expression levels of the TAK1-TAB complex (59). Autophagy is a major catabolic process which is known to degrade and recycle damaged organelles and long-lived cytoplasmic macromolecules (34). Autophagy takes part in the life cycle of HBV under certain conditions (40, 60, 61). Another study reported that HBV infection induced autophagy through the HBx protein or an HBsAg-dependent mechanism (37). Yet another study showed that intracellular HBsAg, but not extracellular HBsAg, induced autophagy in hepatoma cells (38). Lin et al. found that glucosamine promoted HBV replication and HBsAg expression *via* suppressing autophagic degradation (43). The value of LC3II/I increased with HBsAg in a dose-dependent manner in HepG2 cells with or without the treatment of CQ (**Figure 5E**), and HBsAg promoted the puncta formation of LC3B (**Supplementary Figure 3**). Our results are clearly in favor of the notion that HBsAg induces autophagy. Therefore, we repeated the competitive Co-IP experiments in **Figure 5B** with the treatment of MG132 (a proteasome inhibitor) and CQ (an autophagy inhibitor) (**Figures 5C, D**). CQ, but not MG132, inhibited the degradation of TAK1 and TAB2 caused by the expression of HBsAg, and the interference of HBsAg on TAK1-TAB2 interaction disappeared. Endogenous TAK1 and TAB2 were also degraded by HBsAg *via* the autophagy pathway (**Figure 5E**). As mentioned above, HBsAg inhibited the K63 but not K48-linked polyubiquitination of TAB2. Some studies have reported that K48 polyubiquitination chains regulate protein levels by signaling a target protein for degradation by the proteosome, while K63-linked chains regulate processes proteosome-independently such as inflammatory signal transduction, DNA repair, endocytosis, and selective autophagy etc (62). We speculated that autophagy, but not ubiquitin-proteasome system plays a role on TAK1-TAB2 degradation in the presence of HBsAg. We further found that HBsAg promoted the colocalization of exogenous TAK1 and LC3B in HepG2 cells, which was increased upon CQ treatment (**Figures 6A, B**). TAK1 and LC3B colocalized more markedly in HepG2.215 cells than in HepG2 cells with the treatment of CQ (**Figures 6C, D**), as well as in HBV infected HepG2-hNTCP cells (**Figures 6E–G**). TAK1 colocalized with LC3 in HBsAg-positive clinical liver tissues (**Figure 7D**). HBsAg interacts directly with TAK1 as shown in **Figure 2**. It has been reported that there is a direct interaction between HBsAg and LC3 (42) and LAMP1 (43). HBsAg can be secreted either by ER-Golgi, multivesicular, or secreted autophagy (42, 63–65). In light of previous reports and our results, we suggest that HBsAg forms a complex with TAK1 and TAB2, fusing with autophagosome, accelerating the autophagic degradation of TAK1 and TAB2, and reducing the activation of the NF-κB signaling pathway.

**Figure 8:**
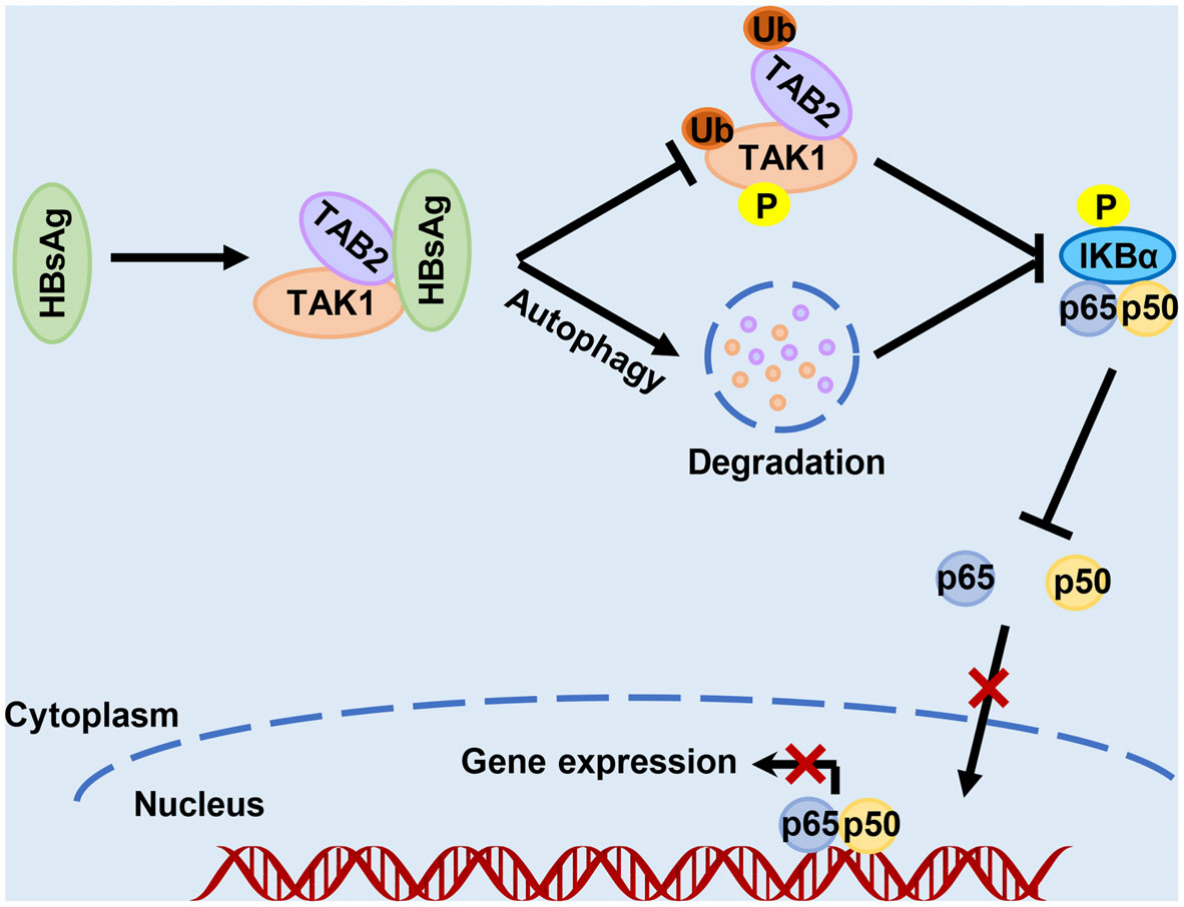
Schematic illustration of how HBsAg interferes with the nuclear factor kappa B (NF-κB) signaling pathway. HBsAg specifically binds to TAK1 and TAB2, suppresses the polyubiquitination of TAK1-TAB2 complex, blocks TAK1 and IKBα phosphorylation, inhibits the NF- κB subunits p50 and p65 translocation from cytoplasm to nucleus, and downregulates the expression of IFNs and inflammatory cytokines. On the other hand, HBsAg interferes the molecular interaction between TAK1 and TAB2 with promoting autophagic degradation, further inhibiting the activation of NF-κB signaling pathways.

In conclusion, as the diagram in **Figure 8** shows, this study revealed a novel molecular mechanism for HBsAg assisting HBV escape from innate immune responses. HBsAg suppresses the activation of NF-κB signaling pathway *via* regulation of post-translational modification of the TAK1 and TAB2 complexes. HBsAg also interacts with TAK1 and TAB2, accelerating the degradation of TAK1 and TAB2 with the help of autophagy, reducing the activation of the NF-κB signaling pathway. These findings provide a new insight into the role of HBsAg in assisting HBV to evade host innate immune responses, which may contribute to the elimination of persistent HBV infection.

## Data Availability Statement

The original contributions presented in the study are included in the article/**Supplementary Material**. Further inquiries can be directed to the corresponding author.

## Ethics Statement

The studies involving human participants were reviewed and approved by the institutional review board of Wuhan University. The patients/participants provided their written informed consent to participate in this study.

## Author Contributions 

All authors meet the criteria for authorship. FD, GX, ZC and YZ designed the study, analyzed the data, wrote and revised the manuscript. FD, YH, CM, CL, and CY performed the experiments. JW and XX contributed to clinical liver and serum specimens. SL and YZ contributed to critical comments and revision of the manuscript. All authors contributed to the article and approved the submitted version.

## Funding

This work was supported by research grants from the National Natural Science Foundation of China (81971494) and the Natural Science Foundation of Hubei Province Innovation Group (2017CFA022). The funding agencies had no role in study design, data collection or analysis, decision to publish, or preparation of the manuscript.

## Conflict of Interest

The authors declare that the research was conducted in the absence of any commercial or financial relationships that could be construed as a potential conflict of interest.

## Abbreviations

Co-IP, coimmunoprecipitation; CQ, chloroquine; GFP, green fluorescence protein; HA, hemagglutinin; HBsAg (HBs), hepatitis B surface antigen; HBV, hepatitis B virus; IB, immunoblotting; IFN, interferon; IkBа, NF-kappa-B inhibitor alpha; IL6, interleukin 6; IP, immunoprecipitation; LC3, microtubule-associated protein 1 light chain 3; NF-κB, nuclear factor kappa B; qRT-PCR, quantitative reverse transcriptase polymerase chain reaction; SEM, standard error of the mean; SeV, Sendai virus; TAB1/2/3, TGF-Beta Activated Kinase 1 Binding Protein 1/2/3; TAK1, TGF-β activated kinase 1; TNFα, tumor necrosis factor alpha; Ub, ubiquitination; VSV, vesicular stomatitis virus.
