# Supplementary material for: Hepatitis B Surface Antigen Suppresses the Activation of Nuclear Factor Kappa B Pathway via Interaction With the TAK1-TAB2 Complex
Source: Front Immunol. 2021 Feb 25;12:618196. doi: 10.3389/fimmu.2021.618196 (PMC7947203; doi:10.3389/fimmu.2021.618196)
Supplement: Supplementary file 1 [file DataSheet_1.docx]

***Supplementary Material***

**1. Supplementary Figures and Tables**

**1.1 Supplementary Figures**

**
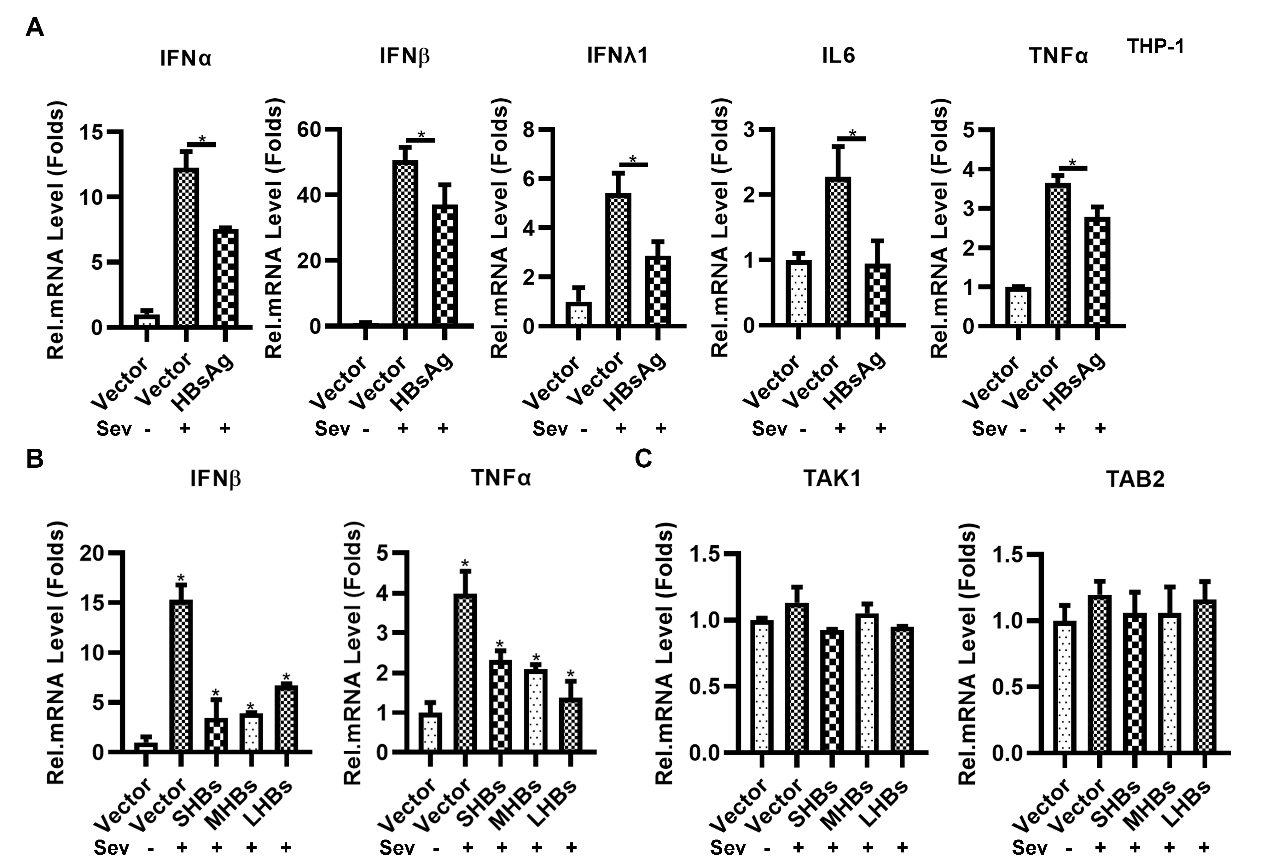
**

**Supplementary Figure 1. (A)** Overexpression of HBsAg downregulated the innate immune responses in macrophages induced with THP-1. Macrophages induced with THP-1 were electro-transfected with 3×Flag-HBsAg or vector plasmids (0.5 μg). At 24 h post transfection, the cells were serum starved for 12 h. cells were infected with SeV (MOI = 1) for 12 h, and then harvested for indicated genes mRNA levels determination by qRT-PCR. (**B** and **C**) Overexpression of all kinds of HBsAg downregulated the innate immune responses (**B**), but makes no difference on the mRNA levels of TAK1 and TAB2 in HepG2 cells (**C**). HepG2 cells were transfected with indicated plasmids (0.5 μg). At 24 h post-transfection, the cells were serum starved for 12 h. cells were infected with SeV (MOI = 1) for 12 h, and then harvested for indicated genes mRNA levels determination by qRT-PCR.

**
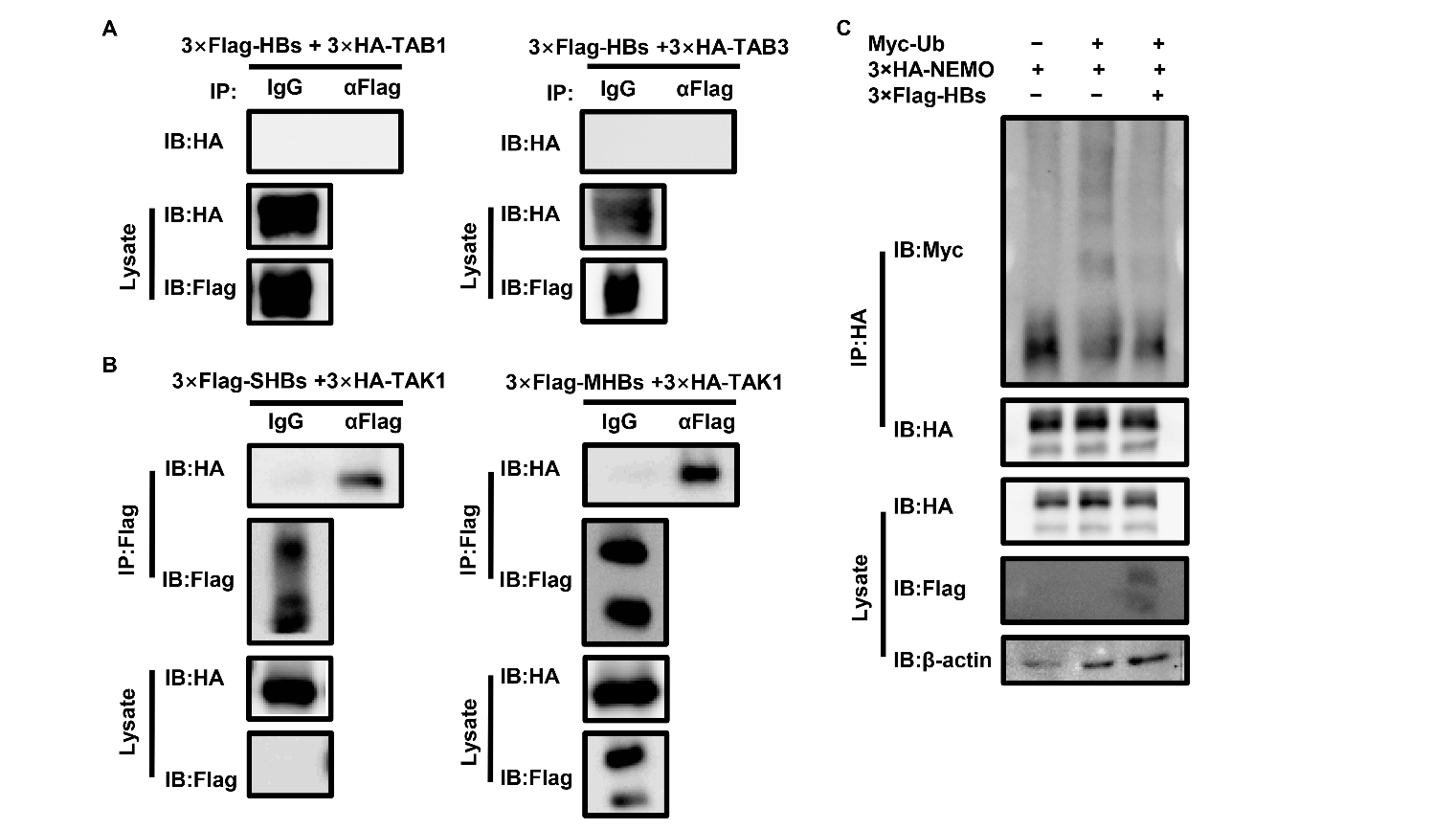
**

**Supplementary Figure 2.** (**A**) HBsAg interact with neither TAB1 or TAB3. 3×Flag-HBs (HBsAg) or vector plasmids were co-transfected into HEK293T cells with 3×HA-TAB1/TAB3, at 48 h post transfection, cells were harvested for co-immunoprecipitation (co-IP) assays. (**B**) TAK1 interacts with both SHBsAg and MHBsAg in vitro. 3×Flag-SHBs/MHBs (SHBsAg/MHBsAg) or vector plasmids were co-transfected into HEK293T cells with 3×HA-TAK1, at 48 h post transfection, cells were harvested for co-immunoprecipitation (co-IP) assays. (**C**) Overexpression of HBsAg inhibits the ubiquitination of NEMO. 3×Flag-HBs (HBsAg) or vector plasmids were co-transfected into Huh7 cells with 3×HA-NEMO and Myc-Ub for 36 h, and then cells were stimulated with virus for 12 h, followed by ubiquitination assay.


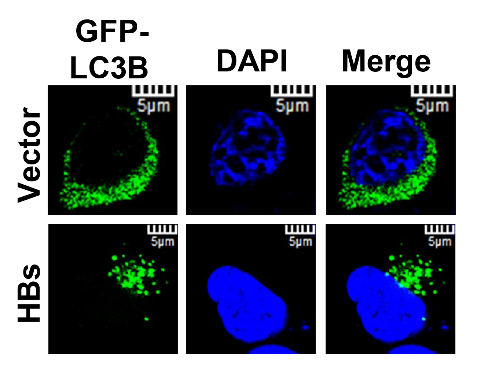


**Supplementary Figure 3.** HBsAg promotes the puncta formation of LC3B. GFP-LC3B were co-transfected into HepG2 cells with HBs expression plasmids (3×Flag-HBs) or empty vector for 48 h; The nuclei were stained with DAPI before observation using confocal microscopy. Scale bar = 5 μm.

**1.2 Supplementary Tables**

**Supplementary Table Ⅰ.** Characteristics of volunteers whose liver tissues or serum were used in this study.

| **Samples** | **Liver** | | **Serum** |
| --- | --- | --- | --- |
| **Characteristic** | **Healthy people (N=10)** | **HBV-infected Patients (N=10)** | **HBV-infected Patients (N=34)** |
| Age (years) | 48.3±14.2 | 50.3±8.9 | 44±14.6 |
| Gender (male/female) | 7/3 | 9/1 | 22/12 |

**Supplementary Table Ⅱ.** List of primers used for qRT-PCR in this study.

| **Target** | **Forward primer (5’ to 3’)** | **Reverse primer (5’ to 3’)** |
| --- | --- | --- |
| IFNα | TTTCTCCTGCCTGAAGGACAG | GCTCATGATTTCTGCTCTGACA |
| IFNβ | AAAGAAGCAGCAATTTTCAGC | CCTTGGCCTTCAGGTAATGCA |
| IFNλ1 | CTTCCAAGCCCACCCCAACT | GGCCTCCAGGACCTTCAGC |
| IL6 | TGGTGGATGTTCCCCCCGAG | TCCTGGGAATACTGGCACGG |
| TNFα | CTTCTCGAACCCCGAGTGAC | ATGAGGTACAGGCCCTCTGA |
| TAK1 | ACTTGATGCGGTACTTTCCA | CTCTGTTGCTTTGCCTGATT |
| TAB2 | CAGCAGGAGCCACAGACAGC | CAAAGATGTAGGAGTATTACGACC |
| GAPDH | AAGGCTGTGGGCAAGG | TGGAGGAGTGGGTGTCG |
| VSV-P | GTGACGGACGAATGTCTCATAA | TTTGACTCTCGCCTGATTGTAC |
| HBV | GGAGGGATACATAGAGGTTCCTTGA | GTTGCCCGTTTGTCCTCTAATTC |
